# De Novo Transcriptomic Characterization Enables Novel Microsatellite Identification and Marker Development in *Betta splendens*

**DOI:** 10.3390/life11080803

**Published:** 2021-08-09

**Authors:** Huapu Chen, Xiaomeng Li, Yaorong Wang, Chunhua Zhu, Hai Huang, Wei Yang, Guangli Li

**Affiliations:** 1 Guangdong Research Center on Reproductive Control and Breeding Technology of Indigenous Valuable Fish Species, Key Laboratory of Marine Ecology and Aquaculture Environment of Zhanjiang, Fisheries College, Guangdong Ocean University, Zhanjiang 524088, China; chenhp@gdou.edu.cn (H.C.); lxm49349@163.com (X.L.); yaorongwang217@126.com (Y.W.); zhu860025@163.com (C.Z.); 2 Key Laboratory of Utilization and Conservation for Tropical Marine Bioresources (Hainan Tropical Ocean University), Ministry of Education, Sanya 572022, China; huanghai74@126.com; 3 Food and Environmental Engineering Department, Yangjiang Polytechnic, Yangjiang 529566, China

**Keywords:** siamese fighting fish, *Betta splendens*, illumina RNA-Seq, gene function annotation, SSR

## Abstract

The wild populations of the commercially valuable ornamental fish species, *Betta splendens*, and its germplasm resources have long been threatened by habitat degradation and contamination with artificially bred fish. Because of the lack of effective marker resources, population genetics research projects are severely hampered. To generate genetic data for developing polymorphic simple sequence repeat (SSR) markers and identifying functional genes, transcriptomic analysis was performed. Illumina paired-end sequencing yielded 105,505,486 clean reads, which were then de novo assembled into 69,836 unigenes. Of these, 35,751 were annotated in the non-redundant, EuKaryotic Orthologous Group, Swiss-Prot, Kyoto Encyclopedia of Genes and Genomes and Gene Ontology databases. A total of 12,751 SSR loci were identified from the transcripts and 7970 primer pairs were designed. One hundred primer pairs were randomly selected for PCR validation and 53 successfully generated target amplification products. Further validation demonstrated that 36% (n = 19) of the 53 amplified loci were polymorphic. These data could not only enrich the genetic information for the identification of functional genes but also effectively facilitate the development of SSR markers. Such knowledge would accelerate further studies on the genetic variation and evolution, comparative genomics, linkage mapping and molecular breeding in *B. splendens*.

## 1. Introduction

The freshwater Siamese fighting fish (*Betta splendens*) is native to southeast Asia and represents one of the labyrinth fishes with the highest commercial value. Similar to several other teleost fishes, *B. splendens* displays unique reproductive and paternal care behaviors and exhibits significant differences in terms of the typical behavior (also known as behavioral typicality) between males and females [1]. During the reproductive process and after oviposition, the males show a higher aggressivity towards invasive conspecies to establish and defend their own territory [2]. Owing to its male-typical behavior and notable attractiveness (e.g., color and scale pattern, shape, diverse fin pattern design and easy culture in poor quality water) [3,4], *B. splendens* is among the most popular aquarium fish species in the ornamental fish industry. Unfortunately, as the commercial breeding and seedling production progresses, it has been reported that the long history of domestication of *B. splendens* has led to the loss of genetic variation of broodstock [3]. Moreover, the wild populations and germplasm resources of this species are now seriously threatened, mainly because of degradation of natural habitat and contamination from artificially bred fish [4]. Therefore, there is an urgent need to strengthen biodiversity conservation and population structure management, much greater efforts must be devoted to protect and improve *B. splendens* germplasm resources.

In aquaculture, population genetics information has been shown to be essential for the strategy formulation of fisheries management and stock improvement. Fifteen years ago, allozyme markers-based population analysis was conducted to quantify genetic variation within and between stocks of hatchery reared *B. splendens* [3]. However, to facilitate the sustainable management of population structures in the wild and in breeding programs in hatcheries, more effective and reliable methods utilizing molecular markers are desperately needed. Among different types of molecular marker technologies, simple sequence repeat (SSR) analysis is strongly recommended for the assessment of population diversity and structure. Thanks to its technical advantages (e.g., locus-specificity, codominance and multiallelic polymorphism), SSR markers are of great importance and have been widely used in genetic analyses [5,6]. To date, the existing studies in *B. splendens* focus mostly on the fields of behavioral ecology [1], pharmacology [7,8], toxicology [9], biological underpinnings of aggressivity [10,11] and genome sequencing [12]; molecular population genetic analysis has been ignored for a long time. Although nine microsatellite markers had been developed from an enriched genomic DNA library [4], the low polymorphism at these microsatellite loci and the scarcity of effective SSR marker resources seriously hamper the progress in population monitoring of fighting fish and an exhaustive survey is still absent. Further researches on population genetics and genomics require much more *Betta*-specific and polymorphic SSR markers.

Currently, the development of SSR markers remains impeded by time and cost constraints. Transcriptome is a small but important part of the genome that contains a large number of coding genes. With enhanced efficiency, transcriptome sequencing based on next generation sequencing (NGS) is widely used to obtain large-scale genetic information. This information can be used to quickly and economically generate large amounts of gene sequence and expression data, especially for non-model species [13,14]. Transcriptome sequencing has been widely applied in the discovery, expression and regulatory analysis of functional genes, as well as the screening of a large number of molecular markers [15]. In the present study, Illumina-based RNA sequencing, transcriptome characterization and expressed sequence tag-simple sequence repeat (EST-SSR) marker development were performed in *B. splendens* for the first time. The main purposes of this study are (i) to enrich genetic data and gene sequence resources for the identification and development of functional genes and (ii) to develop a large number of polymorphic EST-SSR markers. These data will provide support for further researches on population genetics and germplasm conservation.

## 2. Materials and Methods

### 2.1. Animal Material and Sample Collection

All animal experiments were approved by the Animal Research and Ethics Committee of Guangdong Ocean University, Zhanjiang City, China (NIH Pub. No. 85–23, revised 1996). For transcriptomic sequencing, 1-year-old mature *B. splendens* (females, n = 10; males, n = 10) were obtained from a hatchery center (Sanya, Hainan, China). The live fish were decapitated after being anesthetized in an immersion bath containing tricaine methanesulfonate (MS222). Samples, including brain, heart, muscles, liver, kidneys, intestines, spleen, gills and gonads (testes or ovaries) were removed as soon as possible and immediately frozen in liquid nitrogen and stored at −80 °C for RNA extraction. To verify the polymorphism of EST-SSR markers, 30 *B. splendens* individuals were randomly collected from the parent population of the incubation center and the skeletal muscle tissue was removed. Muscle specimens were stored in anhydrous ethanol at −20 °C until genomic DNA extraction.

### 2.2. RNA Extraction and Construction and Sequencing of the cDNA Library

Total RNA was extracted from each tissue of both female and male *B. splendens* using the Trizol kit (Life Technologies, Carlsbad, CA, USA). The extracted RNA was treated by DNase I (TaKaRa Biotech Co., Ltd., Dalian, China) to eliminate genomic DNA. The concentration of total RNA was detected by NanoDrop2000c (Thermo Scientific, Wilmington, DE, USA), utilizing the absorbance at 260 nm, and the purity was assessed by *OD*_260/280_ (acceptable range, 1.8–2.0). The 18S and 28S ribosomal bands stained with ethidium bromide on 0.8% agarose gels were used for the assessments of RNA integrity. After collecting the same amount of RNA samples from different tissues (about 1 μg for each tissue), both male and female RNA samples (about 5 μg for each sex) were sent to Guangzhou Jinyu Biotechnology Co., Ltd. (Guangzhou, Guangdong, China) for cDNA library construction and sequencing. The Oligo-dT Beads Kit (Qiagen, Hilden, Germany) was used to purify mRNA and cDNA libraries were constructed according to the Illumina RNA sequencing protocol. Two cDNA libraries were sequenced on an Illumina HiSeq™ 2000 sequencing platform (Illumina, Inc., San Diego, CA, USA) and paired-end (PE) reads with a length of 125 bp were generated.

### 2.3. De Novo Assembly

SOAPnuke v1.5.0 was used with the parameters ‘-l 10 -q 0.5 -n 0.05 -p 1 -i’ to control the quality of the original sequencing data. To produce high quality data, the original read was filtered by removing the sequence of adapters, ambiguity sequences (N) greater than 10% and low-quality sequences (quality value < 20) greater than 20%. Then, the Trinity RNA-Seq Assembler (version: r20140717, http://trinityrnaseq.sourceforge.net (accessed on 15 June 2015)) was used with default parameters to assemble high-quality clean reads [15]. Firstly, a clean read was assembled by Inchworm using the greedy k-mer method, resulting in a set of linearly overlapping groups. Then, Chrysalis built rich contigs into the de Bruijn graph with k-1 overlaps. Finally, the fragmented de Bruijn graphs were trimmed, compacted and reconciled to final linear transcripts using Butterfly. The redundant final linear transcripts were removed and the longest transcripts were defined as unigenes [16].

### 2.4. Annotation and Classification

For functional annotations, the BLAST 2.2.26+ software (NCBI, Rockville, MD, USA) was used to perform sequence alignment on the public database and the E-value cut-off 1E-5 was used. Sequence homology of all assembled single genes was assessed with BLASTx and protein databases, such as the National Center for Biotechnology Information (NCBI) non-redundant (NR), EuKaryoticOrthologous Group (KOG), Swiss-Prot and Kyoto Encyclopedia of Genes and Genomes (KEGG), were used. Sequences with the highest similarity score in the database were defined as the functional annotation of related unigenes. The functional results of Gene Ontology (GO) were obtained using the Blast2GO software (BioBam Bioinformatics, Cambridge, MA, USA) [17] and then classified [18]. The KOBAS v2.0 software(Beijing University, Beijing, China) was used for path classification analysis in KEGG path annotation [19]. In addition, the assembled single genes were compared with the KOG database to predict and classify possible functions.

### 2.5. SSR Loci Search and Primer Design

SSR locus detection was performed on assembled single gene sequences using the Perl program MIcroSAtellite (MISA, http://pgrc.ipk-gatersleben.de/misa/ (accessed on 22 July 2016)) [20]. Single nucleotide repeats were excluded from this study because of the difficulty to distinguish true single nucleotide repeats from polyadenosylated products and single nucleotide extension errors resulting from sequencing. Dinucleotide, trinucleotide, tetranucleotide, pentanucleotide and hexanucleotide SSR motifs were identified using at least six, five, four, four and four consecutive repeats, respectively. Primer pairs on both sides of each SSR site were designed using the Primer 5.0 software [21].

### 2.6. SSR Polymorphism Examination

Genomic DNA was extracted from muscle samples using the marine animal tissue genomic DNA kit (Tiangen Biotech, Beijing, China) following the manufacturer’s instructions. The quality and quantity of the extracted DNA were determined using a NanoDrop2000 spectrophotometer (Thermo Scientific, Willmington, DE, USA) and 1.0% agarose gel electrophoresis. The DNA samples were diluted with ddH_2_O to a final concentration of 20 ng/μL and stored at −20 °C for PCR analysis. PCR was performed on the C1000™ Thermal Cycler (Bio-Rad, Hercules, CA, USA) with a total volume of 10.0 μL. Each reaction tube contained 1.0 μL of DNA template (20 ng), 0.2 μL of primers (10 μmol/L), 5.0 of μL 2×EasyTaq PCR SuperMix (Invitrogen, CA, USA) and 3.6 of μL ddH_2_O. The PCR cycle conditions were as follows: initial denaturation at 94 °C for 5 min, followed by 30 cycles of 45 s at 94 °C, 40 s at the annealing temperature and 40 s at 72 °C and a final extension at 72 °C for 5 min. PCR products were separated on an 8.0% non-denaturing polyacrylamide gel and allele sizes were estimated according to the pBR322 DNA/MspI marker (Tiangen Biotech, Beijing, China). The number of alleles (*N*_a_), expected heterozygosity (*H*_e_), observed heterozygosity (*H*_o_) and polymorphism information content (*PIC*) for each SSR locus were calculated by the PowerMarker v3.25 software [22].

## 3. Results

### 3.1. Sequencing and Assembly

**Figure 1:**
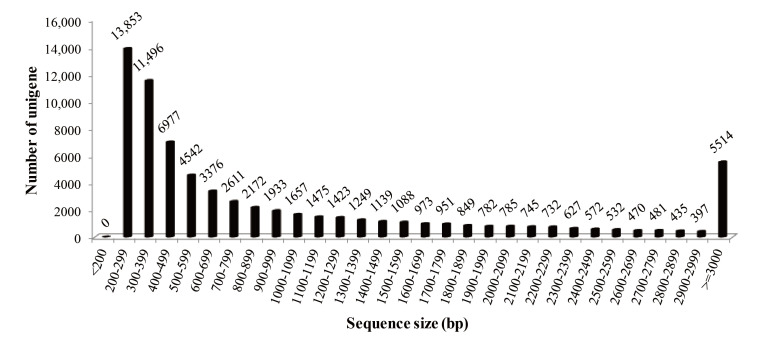
Sequence size distribution of assembled unigenes derived from *Betta splendens* transcriptome.

Male and female cDNA libraries were sequenced using Illumina sequencing technology. The main steps and tools used for bioinformatics analysis are shown in Supplementary Figure S1. The original sequencing data were uploaded to the NCBI Sequence Read Archive (SRA) database and the male and female accession numbers were SRX2598247 and SRX2598248, respectively. A total of 108,416,100 raw PE reads (13.55 GB of sequencing data) were obtained. The quality control of original data produced 105,505,486 clean PE reads (53,062,092 for female and 52,443,394 for male), which is equal to 13.19 GB of sequencing data. The Q20 percentage of clean data was 97.315% and the GC content was 50.635% (Table 1). These high-quality clean reads were then assembled into 69,836 unigenes with an average length of 1093.52 bp and an N50 length of 2040 bp, representing 76.37 Mbp sequences (Table 1). The sequence length distribution of all assembled genes ranged from 228 bp to 20,412 bp. Exactly, 37,510 (53.71%) unigenes were >500 bp in length, 22,876 (32.76%) unigenes were >1000 bp in length and 11,290 (16.17%) unigenes were >2000 bp in length (Figure 1).

### 3.2. Functional Annotation

**Figure 2:**
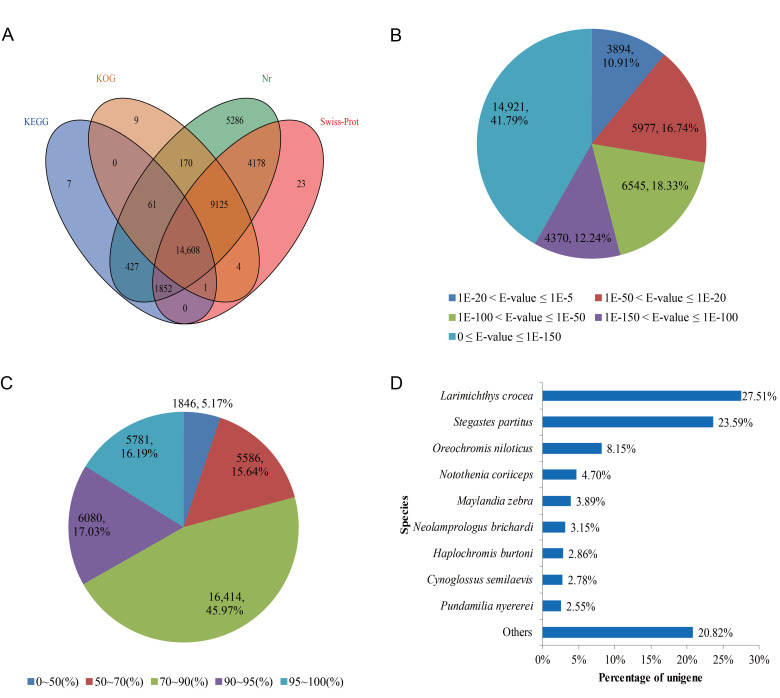
Summary of unigene assembly and annotation statistics. (**A**) Venn diagram of the non-redundant (NR) unigene annotated result, EuKaryotic Orthologous Group (KOG), Swiss-Prot and Kyoto Encyclopedia of Genes and Genomes (KEGG) databases. The number in each color block represents the number of unigenes for single or multiple database annotations. (**B**) E-value distribution of BLAST search against the natural resource database for each unique sequence (E-value 1E-5). (**C**) The identity distribution of BLAST was specific to each unique sequence. (**D**) The species distribution of BLAST was consistent with that of assembled unigenes (E-value 1E-5), showing the top nine matching species.

All unigene sequences were annotated by BLASTx search against public databases. A total of 35,751 (51.19%) unigenes were annotated in at least one of the queried databases. The transcripts of these annotations were remarkably similar to the proteins in the database, with 35,707 (51.13%) from NR, 29,791 (42.66%) from Swiss-Prot, 23,978 (34.33%) from KOG and 16,956 (24.28%) from KEGG. Furthermore, 14,608 (24.28%) unigenes were annotated in all databases (Figure 2A). The E-value distribution of Blast analysis revealed that 72.36% of unigenes showed significant homology (below 1E-50) in the NR database and 16.74% of the homologous sequences ranged between 1E-20 and 1E-50 (Figure 2B). Moreover, 79.19% and 33.22% of sequences had more than 70% and 90% similarity, respectively (Figure 2C). Further analysis of the matching sequences yielded homologous genes from 304 species. Of these, 27.51% of the annotated unigenes had the highest homology with genes from *Larimichthys crocea*, followed by *Stegastes partitus* (23.59%), *Oreochromis niloticus* (8.15%), *Notothenia coriiceps* (4.70%), *Maylandia zebra* (3.89%) and *Neolamprologus brichardi* (3.15%). These six species accounted for more than 70% of the annotated unigenes (Figure 2D).

### 3.3. GO, KEGG Pathway and KOG Classification

**Figure 3:**
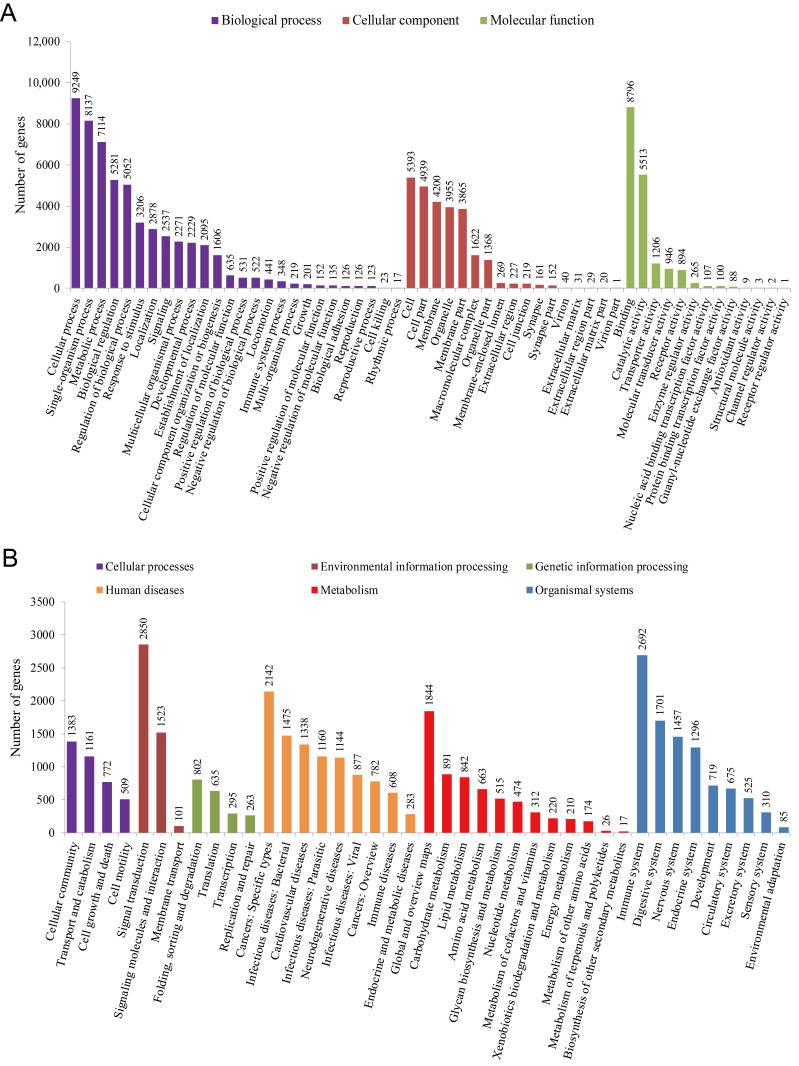
Functional classification of single genes annotated in the GO and KEGG pathways. (**A**) A total of 16,954 unigenes showed significant similarities to homologous genes in the GO database, which were grouped into the following three broad categories: cellular components, molecular functions and biological processes. (**B**) KEGG pathways can be divided into six major categories: genetic information processing, metabolism, cellular processes, biological systems, environmental information processing and human diseases.

To understand the function of unigenes, Blast2GO was used to assign GO terms to each sequence. WEGO tools were then used to perform GO classification and visualization. In total, 16,954 (24.28%) unigenes, annotated in the GO database with one or more GO terms, were assigned to 56 level-2 functional groups (Figure 3A, Supplementary Table S1). Of these, ‘biological process’ (55,254; 55.43%) was found to be the largest level-1 category, followed by ‘cellular component’ (26,491; 26.58%) and ‘molecular function’ (17,930; 17.99%). In the ‘biological process’ category, the level-2 GO terms ‘cellular process’ (9249; 9.28%) and ‘single-organism process’ (8137; 8.16%) were found to be prominently represented. In the category ‘cellular component’, ‘cell’ (5393; 5.41%) and ‘cell part’ (4939; 4.95%) were highly represented. In the category ‘molecular function’, ‘binding’ (8796; 8.82%) and ‘catalytic activity’ (5513; 5.53%) accounted for a significant proportion. 

To study the activated biological pathways of *B. splendens*, unigene KEGG pathways were analyzed. The results showed that 16,956 (24.28%) unigenes were classified into six major categories, including a total of 240 different KEGG pathways and 35,751 genes (Figure 3B, Supplementary Table S2). Of these, the largest category, ‘human diseases’, contained 9809 (27.44%) KEGG-annotated genes, followed by ‘organismal systems’ (9460; 26.46%), ‘metabolism’ (6188; 17.31%), ‘environmental information processing’ (4474; 12.51%), ‘cellular processes’ (3825; 10.70%) and genetic information processing (1995; 5.58%). Of the top 10 pathways, 55.53% of sequences were involved in ‘global and overview maps’, ‘cancers: overview’ and ‘signal transduction’. The rest of the sequences were related to pathways such as ‘signaling molecules and interaction’, ‘cell motility’, ‘cellular community’ and ‘development’. 

**Figure 4:**
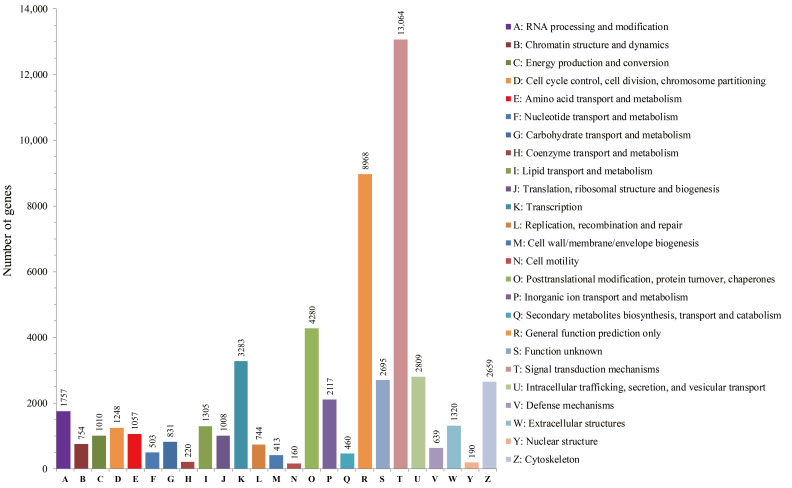
Annotated eukaryotic homologous group (KOG) classification of unigenes. Of the 35,707 NR matched unigenes, 23,978 (67%) were classified into 25 KOG categories.

In addition, function prediction and classification analysis of all unigenes were performed by retrieving the predicted unigene coding sequences from the KOG database. A total of 23,978 unigenes were successfully annotated and grouped into 25 subclasses (Figure 4, supplementary Table S3). The largest orthology cluster ‘signal transduction mechanisms’ accounted for 24.42% (13,064) of the total annotations, followed by ‘general function prediction only’ (8968; 16.76%), ‘posttranslational modification, protein turnover, chaperones’ (4280; 8.00%), ‘transcription’ (3283; 6.14%), ‘intracellular trafficking, secretion and vesicular transport’ (2809; 5.25%), ‘function unknown’ (2695; 5.04%), ‘cytoskeleton’ (2659; 4.97%) and ‘inorganic ion transport and metabolism’ (2117; 3.96%). These functional labels and classifications will provide abundant resources for gene mining and functional analysis.

### 3.4. SSR Loci Identification and Polymorphism Verification

**Figure 5:**
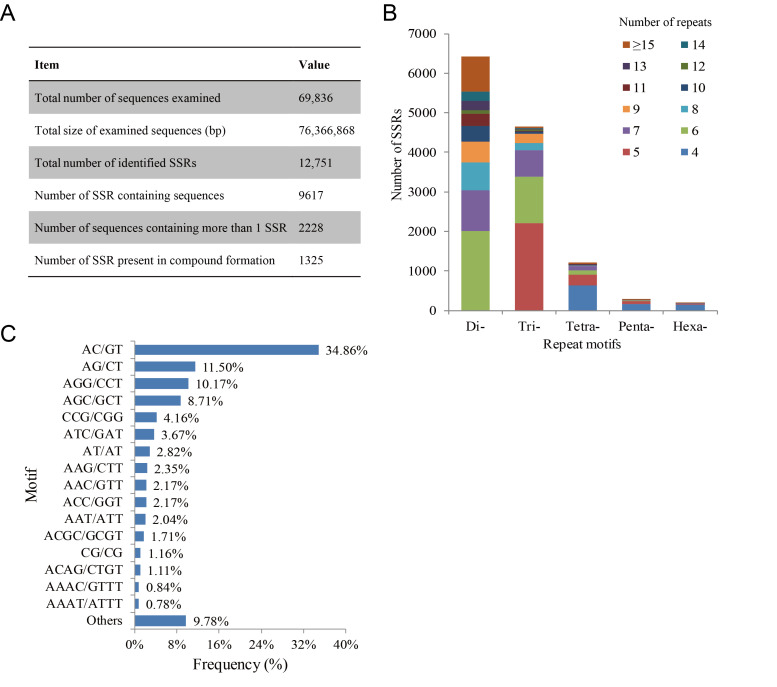
Identification of SSR loci in the transcriptome of *B. splendens*. (**A**) Statistics of SSR locus detection. (**B**) Distribution of the number of repeats of dinucleotide, trinucleotide, tetranucleotide, pentanucleotide and hexanucleotide repeats. (**C**) Frequency distribution of repeated pattern classification types showing the frequency of occurrence of major pattern types.

A total of 9617 (13.77%) unigene sequences containing 12,751 potential SSR loci were detected by the MISA software and 1809 sequences contained more than one SSR locus (Figure 5A). The distribution density of SSRs in the transcriptome was one locus per 5.99 kb. The most abundant SSRs were di-nucleotide repeats (6418; 50.33%), followed by tri-nucleotide repeats (4657; 36.52%), tetra-nucleotide repeats (1209; 9.48%), penta-nucleotide repeats (279; 2.19%) and hexa-nucleotide repeats (188; 1.47%) (Figure 5B). The copy number of different repeat units ranged from 4 to 41. The frequency distribution of motif sequence types was further analyzed. In dinucleotide and trinucleotide repeat motifs, AC/GT (4445; 34.86%), AG/CT (1466; 11.50%) and AGG/CCT (1297; 10.17%) repeats were the three main types of the transcriptome of *B. splendens* (Figure 5C).

For the development of microsatellite markers, 7970 (62.50%) sequences containing SSR loci enabled the design of primers. The information of SSR primers is shown in Supplementary Table S4. To assess the genetic polymorphism of these markers, 100 SSR primers were randomly selected for primer synthesis and PCR validation. The results showed that 53 primer pairs successfully generated stable and repeatable target amplification products using genomic DNA. Using these 53 primer pairs, a *B. splendens* population containing 30 individuals was analyzed. A total of 34 SSR loci were found to be monomorphic and 19 were polymorphic. A total of 65 alleles were amplified from these polymorphic loci and the number of alleles per locus ranged from 2 to 5, with an average of 3.42 alleles. The observed *(H*_o_) and expected heterozygosity (*H*_e_) values ranged from 0.167 to 0.933 and from 0.430 to 0.772, with average values of 0.575 and 0.618, respectively. The polymorphic information content (*PIC*) values ranged from 0.357 to 0.617, with a mean value of 0.534 (Table 2).

## 4. Discussion

### 4.1. Characterization of B. splendens Transcriptome

Transcriptome sequencing is the preferred method for obtaining large-scale functional gene sequences and enriching the genetic resource pool of non-model species rapidly and economically [23]. To obtain a representative transcriptome of *B. splendens*, different tissue samples were collected for RNA isolation. The extracted total RNAs were pooled in equal amounts for the construction of male and female cDNA libraries. This multi-tissue strategy has been widely used in RNA-seq studies for teleosts [16,24,25,26]. For high-throughput short-read sequencing, high-quality assembly facilitates post-transcriptional annotation, gene identification, comparative genomics and other analyses. Compared with other de novo assembly tools for NGS technologies (e.g., Newbler [27], iAssembler [28] and CLC Genomics Workbench [29]), Trinity gives more satisfactory results, as it can provide a unified and better solution for the reconstruction of transcriptomes without requiring a reference genome [15,30]. According to most of published transcriptome sequencing data, the quality of de novo assembly is primarily assessed by the length distribution of transcripts [30]. In the present study, the N50 length was 2040 bp and more than half of the unigenes were larger than 500 bp in length, with an average length of 1093 bp (Table 1, Figure 1). Similar results have been reported in Trinity-assembled transcriptomes of other teleost fishes, including *Tachysurus fulvidraco* (1611 bp) [31], *Trachinotus ovatus* (1179 bp) [24] and *Scatophagus argus* (906 bp) [16]. The results strongly suggest that the assembly of *B. splendens* transcriptome is effective and accurate.

It has been demonstrated that longer assembly sequences can provide more information for further gene studies and facilitate the identification of molecular markers. In this study, the BLAST matching rate of unigenes was significantly improved between 200–300 bp (32.82%) and 1100–1200 bp (80.74%) in length. However, query sequence length is critical for determining the importance of a BLAST hit. The proportion of unigenes with significant BLAST score increased sharply from 200–500 bp to 500–1500 bp. In summary, for longer assembly sequences, a larger proportion of matched sequences was found in the database, which was consistent with other analysis results of next generation transcriptome sequencing [16,32,33]. In terms of function prediction, 51.19% of unigenes could be matched with homologous sequences by public database search (Figure 2A), indicating successful annotation of transcriptome sequences. However, about half of the transcripts (48.81%) were not annotated to any sequence in the query database. Previous studies have shown that such a high percentage of unannotated sequences is usually caused by untranslated regions of mRNA, misassembled chimeric sequences, unconserved regions of proteins, or new genes [26]. Unsurprisingly, low annotation rates seem to be more generally observed in non-model animals whose genomic sequence data are unpublished, especially aquatic species [24,26,34].

### 4.2. SSR Characterization and Marker Validation

Our study, as the first report of the comprehensive identification, distribution analysis and comparison of SSRs in *B. splendens* transcriptome, found that 9617 (13.77%) unigenes were identified as SSR-containing sequences (Figure 5A). The number of SSR loci (12,751) in these sequences is almost equal to previous reports in teleost fish, such as *Bagarius yarrelli* (14,812) [35] and *Megalobrama amblycephala* (10,877) [36]. Meanwhile, the proportion of SSR-containing transcripts is similar to that in *S. argus* [16] and higher than that in *M. amblycephala* (5.0%) [37], but far less than that in *Paralichthys olivaceus* (22.14%) [38], *Sander lucioperca* (29.0%) [39] and *T. fulvidraco* (49.0%) [40]. There are many possible explanations for this difference, including different kinds of nature and potential artifacts from using different software tools. Among the single genes containing SSR loci, 2228 sequences contained more than one SSR and 255 sequences contained more than two SSRs. Unevenness in SSR distribution has also been found in transcriptome SSR development in other fishes, such as *S. argus* [16], *Acipenser sinensis* [41] and *Carassius auratus* [42]. In the transcriptome of *B. splendens*, one SSR was found per 5.99 kb. The distribution density of microsatellites in this fish was similar to that of other teleosts, such as *T. ovatus* [24] and *Salmo trutta* m. *trutta* [29]. In addition, we discovered that dinucleotide (AC/GT and AG/CT) and trinucleotide repeats (AGG/CCT) were the most abundant SSR motifs (Figure 5B,C). Similar results have been obtained by transcriptome analyses of diverse fish species, including *T. ovatus* [24], *Scophthalmus maximus* [26], *S. argus* [16], *B. yarrelli* [35] and *S. trutta* m. *trutta* [29]. In general, this consistency suggests that the distribution of microsatellites in teleost fish is conserved and the dominant repeating types of SSR are usually primitive.

In this study, a total of 7970 EST-SSR markers were developed and approximately half of randomly selected primers could be successfully amplified. The ratio (53%) of correctly amplified microsatellite markers investigated in this study is significantly lower than that of previous studies (> 90%) [4,43], in which the genomic SSRs were derived from SSR-enriched genomic libraries or random genomic sequences. The remaining half of these primer pairs might have been mismatched and, in such cases, some EST-SSRs could not amplify the targets correctly because of the presence of large introns in the target amplicon, or because the primers were across splice sites. Moreover, nearly 20% of the primers specifically amplified the target PCR product and showed polymorphism (Table 2), indicating that the development of large-scale SSR markers for *B. splendens* is successful. The polymorphism rate of these isolated EST-SSRs in this study was lower than that of other fish species, such as *Carassius auratus*, *Cynoglossus semilaevis* and *Cyprinus carpio*, in which the ratios of polymorphic markers reached 30.2%, 31.0% and 42.0%, respectively [42,44,45], perhaps because the tested individuals came from the same *Betta* fish hatchery. Thus, more polymorphic microsatellites would be developed if geographically remote populations are used for polymorphic verification.

From a genetic perspective, the key parameters (e.g., *H*_o_, *H*_e_ and *PIC*) can reflect the genetic diversity and inheritance patterns of a population from multi-angles [46]. It is generally considered that *PIC* > 0.5 denotes a high polymorphism rate, 0.25 < *PIC* < 0.5 denotes a moderate polymorphism rate and *PIC* < 0.25 denotes a low polymorphism rate [47]. In the present research, the 19 polymorphic SSR loci distinguished the 30 *B. splendens* individuals with an average of 3.42 alleles per locus and an average *PIC* value of 0.534 (Table 2), clearly indicating a high level of polymorphism. This observation is largely consistent with the features of genomic SSR loci in a previous study by Chailertrit et al. [4]. From the comparison, we observed decreased values of the parameters such as the mean number of alleles (3.42 in this study vs. 3.89 in Chailertrit et al.) and average effective number of alleles (2.09 vs. 2.30), while the mean values of observed heterozygosity (0.575 vs. 0.39) and expected heterozygosity (0.618 vs. 0.50) were relatively higher in our study. On the other hand, comparison of our results with previous findings showed that the average values of observed heterozygosity, expected heterozygosity and *PIC* obtained in this study were comparable with or even higher than that of other SSR-based studies in fish species [26,35,48,49,50]. Taken together, the SSR markers developed in the present study are the most abundant polymorphic SSR loci so far, which could serve as an effective molecular tool in further genetic researches in *B. splendens*.

To date, a large number of successful cases have supported the approach of using transcriptome data to predict SSR loci and develop feasible markers [35,39,40,48]. These efforts inspire the advancement of the techniques available for aquaculture germplasm characterization and improvement. SSR markers have extensively been employed for the assessment of genetic diversity and variation, analysis of population structure and fingerprinting for relatedness among individual and populations [51], which can greatly help breeders and germplasm managers prioritize broodstock populations for establishing core collections. Research on the development of SSR markers in this work is just the start. Follow-up studies of genetic analysis will be conducted to quantify genetic variations within and between stocks of hatchery reared *B. splendens* and the difference in population diversity between the natural and captive populations will also be illustrated. In addition, transcriptome or EST-based SSRs (also known as genic SSRs) occur mainly in the coding regions of annotated genes and are important for the determination of functional genetic variation [29]. Compared with genomic SSRs, genic SSR markers are considered to be linked with the loci of economic phenotypes and have become a powerful resource for the application of genetic improvement in aquaculture species. They have been widely used in genetic mapping, comparative and functional genomics, map-based gene cloning and marker-assisted selection (MAS) [50]. Evidently, the potential EST-SSRs identified in the *B. splendens* transcriptome provide a rich reservoir for the development of polymorphic markers in breeding lines, which could significantly increase the efficiency of the related population genomics studies, such as marker-trait association analysis, construction of genetic maps, quantitative trait loci (QTL) mapping and so on.

## 5. Conclusions

An informative transcriptomic dataset containing 69,836 unigene sequences was generated from *B. splendens* tissue samples using Illumina paired-end sequencing. Of these, 35,751 transcripts were successfully annotated by BLAST searches against public protein databases. A total of 12,751 candidate EST-SSR markers were identified and 7970 PCR primers were designed. One hundred randomly selected primer pairs were validated and 19 loci were demonstrated to be polymorphic. These analyses provide expanded insights into the *B. splendens* transcriptome profile and distribution pattern of SSR loci, which will accelerate the identification of functional genes and investigations on the genetic variation of fighting fish. The SSR marker resource with relatively high degrees of polymorphism would be helpful for future studies on genetic diversity and evolution, comparative genomics, linkage mapping and molecular breeding in *B. splendens*.

## Figures and Tables

**Table 1:** Statistical summary of sequencing and assembly of *Betta splendens* transcriptome.

Sequencing				Assembly	
Item	Female Library	Male Library	Total	Item	Value
Raw reads	54,600,768	53,815,332	108,416,100	Unigenes	69,836
Raw bases (bp)	6,825,096,000	6,726,916,500	13,552,012,500	GC content (%)	50.66
Clean reads	53,062,092	52,443,394	105,505,486	Total length (bp)	76,366,868
Clean bases (bp)	6,632,761,500	6,555,424,250	13,188,185,750	N50 length (bp)	2040
–	–	–	–	Mean length (bp)	1093.52

**Table 2:** Characterization of 19 polymorphic SSR loci in 30 *B. splendens* individuals.

SSR ID	Locus		Primer Sequences (5′-3′)	Repeat Motif	Allele Size (bp)	*T*_a_ (°C)	Accession No.	*N*_a_	*N*_E_	*H*_o_	*H*_e_	*PIC*	*P-HWE*
BsSSR008	Unigene0061796	F	GATTCGGACTACGAGCTGGA	(CA)_14_	187	60	MZ615708	4	2.224	0.867	0.642	0.569	0.2670
		R	CACATGAAGCTTAGTGGGGG										
BsSSR010	Unigene0009720	F	CTGGAGAACACGCAGTACGA	(GGACCA)_4_	276	55	MZ615709	3	2.033	0.367	0.633	0.544	0.1735
		R	TTCGGATCAGTGTCATGAGG										
BsSSR014	Unigene0028390	F	GTGACGATGGAGAAAACGGT	(GCC)_6_	149	58	MZ615710	3	2.400	0.833	0.657	0.570	1.0000
		R	GCTTTTCTAGGTTTGCACGG										
BsSSR019	Unigene0050924	F	TCAGAAGAAAGAGGGACGGA	(AC)_8_	280	56	MZ615711	3	2.020	0.700	0.595	0.513	0.0861
		R	GAGAGAGTCCAACCTGCACA										
BsSSR020	Unigene0015325	F	AAGCAGCAACACACGAACAG	(TG)_10_	128	56	MZ615712	3	1.212	0.233	0.430	0.357	0.0364
		R	TTGATTCTCTGCACGCTGTC										
BsSSR023	Unigene0021148	F	ACACAACAGTCTCCCTTCGC	(CGCCA)_5_	203	58	MZ615713	5	2.727	0.724	0.682	0.617	0.1483
		R	GGGGGACAATGGGAGAAATA										
BsSSR031	Unigene0041043	F	AGGAGAGAGTGAGACAGCGG	(AGC)_6_	239	56	MZ615714	3	1.830	0.800	0.615	0.527	0.2250
		R	CTGGAAAACAAGGCAGAAGC										
BsSSR032	Unigene0066200	F	GGCAGCTAAACAACCTCCAG	(TA)_6_	246	56	MZ615715	2	1.593	0.367	0.508	0.375	1.0000
		R	CAGGAGCAGCAGACTTTTCC										
BsSSR039	Unigene0010115	F	GGTCCAAACACAAACCCATC	(GT)_10_	236	54	MZ615716	4	2.053	0.733	0.632	0.556	0.1031
		R	GTGCTTCATGCTTGTGCATT										
BsSSR042	Unigene0013687	F	GGATACAATGAAGGAGCGGA	(CA)_9_	239	58	MZ615717	2	1.112	0.367	0.499	0.371	0.0011*
		R	GCCTGTATTTGCATGTGGTG										
BsSSR043	Unigene0049069	F	TTCCGTTTCCTGGACTTGAC	(TTC)_5_	243	55	MZ615718	2	1.365	0.400	0.506	0.374	0.1162
		R	GGATGACAGTCCCTGAGAGC										
BsSSR044	Unigene0012215	F	GGGTTTGCTCCAGTGATTGT	(GT)_9_	262	55	MZ615719	4	3.200	0.400	0.617	0.541	0.0422
		R	GTCCACAAGCTTCCCGAATA										
BsSSR051	Unigene0059934	F	GTGATATCCTGTCACCGCCT	(ATT)_8_	131	55	MZ615720	4	2.693	0.700	0.671	0.591	0.1483
		R	GACCAAATTAGCAGGGACGA										
BsSSR053	Unigene0003140	F	GTTCGGTGGCAGGGTATAAA	(TG)_16_	194	58	MZ615721	4	1.626	0.367	0.607	0.541	0.0001*
		R	ATGCTTCCTACTGCCCTGTG										
BsSSR068	Unigene0022064	F	ATGAGAGGAGGAGCAGCAAA	(TGCAGC)_4_	234	60	MZ615722	4	2.173	0.400	0.724	0.658	0.1039
		R	GCCTTTGGAAGTGAGACAGG										
BsSSR069	Unigene0008534	F	TAAGAGCCCAGGTTTTCACG	(CAG)_7_	265	55	MZ615723	4	2.441	0.733	0.718	0.652	0.4257
		R	GGCATGTCCTTGATGAGGTT										
BsSSR077	Unigene0033389	F	GGCTGATTCACCCCAAATAC	(TG)_7_	256	53	MZ615724	3	2.390	0.933	0.651	0.568	0.0076*
		R	TTCCTTTGCATTGCTCACAG										
BsSSR079	Unigene0039100	F	CGGAAGCAGCAGTCCTACAT	(GCG)_7_	234	57	MZ615725	3	1.540	0.833	0.594	0.495	0.5415
		R	CTGCTGCAGCTCTTTCCTCT										
BsSSR081	Unigene0006030	F	GTGCGTAAAGCCGAAAGAAG	(GGC)_5_	220	55	MZ615726	5	3.102	0.167	0.772	0.720	0.0692
		R	GTGAGGAGACACCGACTGCT										

Note: *T*_a_, annealing temperature; *N*_a_, number of alleles; *N*_E_, effective number of alleles; *H*_o_, observed heterozygosity; *H*_e_, expected heterozygosity; *PIC*, polymorphic information content; *P-HWE*, *P* value of Hardy–Weinberg equilibrium. * Significant departures from Hardy–Weinberg equilibrium (HWE) (*p* < 0.01).

## Data Availability

The data presented in this study are available on request from the corresponding author. The data are not publicly available due to the agreement with funding bodies.

## Supplementary Materials

The following are available online at https://www.mdpi.com/article/10.3390/life11080803/s1. Table S1: GO annotation results of ungenes in the transcriptome of *B. splendens*, Table S2: KEGG pathway analysis for the transcriptome of *B. splendens*, Table S3: The functional classification of the KOG classes for the transcriptome of *B. splendens*, Table S4: All SSRs primer information were derived from unigenes of the *B. splendens*, Figure S1: The main steps and tools used for bioinformatics analysis.
