# Supplementary figures and images for: De Novo Transcriptomic Characterization Enables Novel Microsatellite Identification and Marker Development in Betta splendens
Source: Life (Basel). 2021 Aug 9;11(8):803. doi: 10.3390/life11080803 (PMC8400612; doi:10.3390/life11080803)

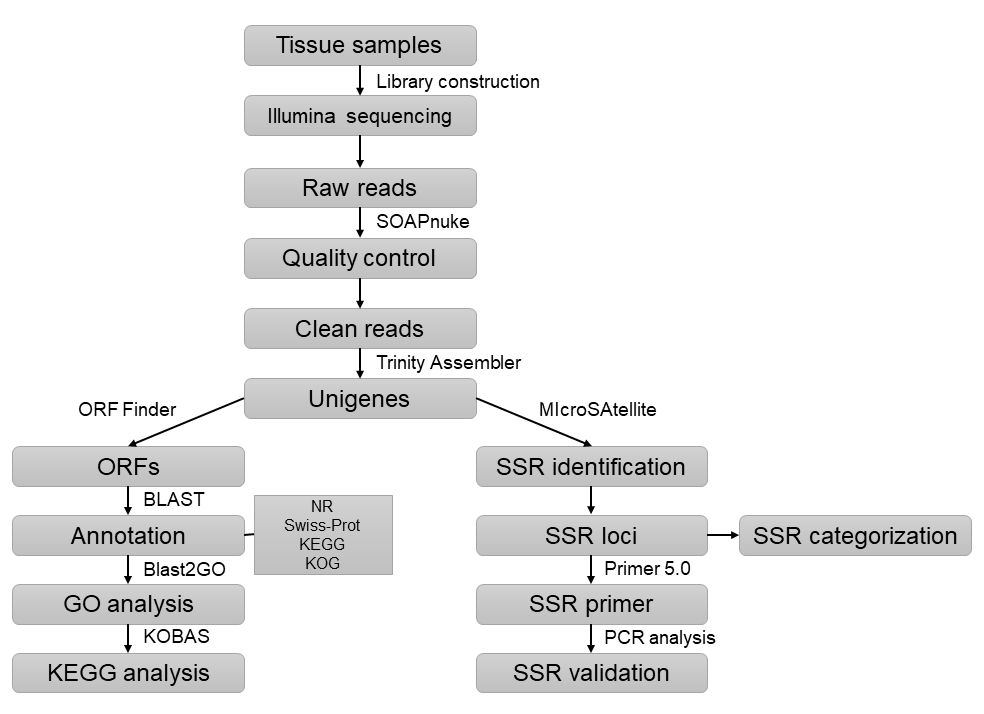

Supplement: Supplementary file 1 [file life-11-00803-s001.zip › Figure S1.tif]
